# Supplementary material for: Prematurely terminated intron-retaining mRNAs invade axons in SFPQ null-driven neurodegeneration and are a hallmark of ALS
Source: Nat Commun. 2022 Nov 22;13:6994. doi: 10.1038/s41467-022-34331-4 (PMC9681851; doi:10.1038/s41467-022-34331-4)
Supplement: Supplementary file 1 — Supplementary Information [file 41467_2022_34331_MOESM1_ESM.pdf]

Supplementary Information for

Prematurely terminated intron-retaining mRNAs invade axons in SFPQ null-driven neurodegeneration and are a hallmark of ALS

Richard Taylor*, Fursham Hamid, Triona Fielding, Patricia M. Gordon, Megan Maloney, Eugene V. Makeyev, Corinne Houart*
Correspondence to: richard.taylor@kcl.ac.uk, corinne.houart@kcl.ac.uk

This file includes:

Supplementary Figs. 1 to 12
Supplementary Tables 1 and 2

Other Supplementary Materials for this manuscript include the following:

Supplementary Data 1 to 10

Supplementary Figures

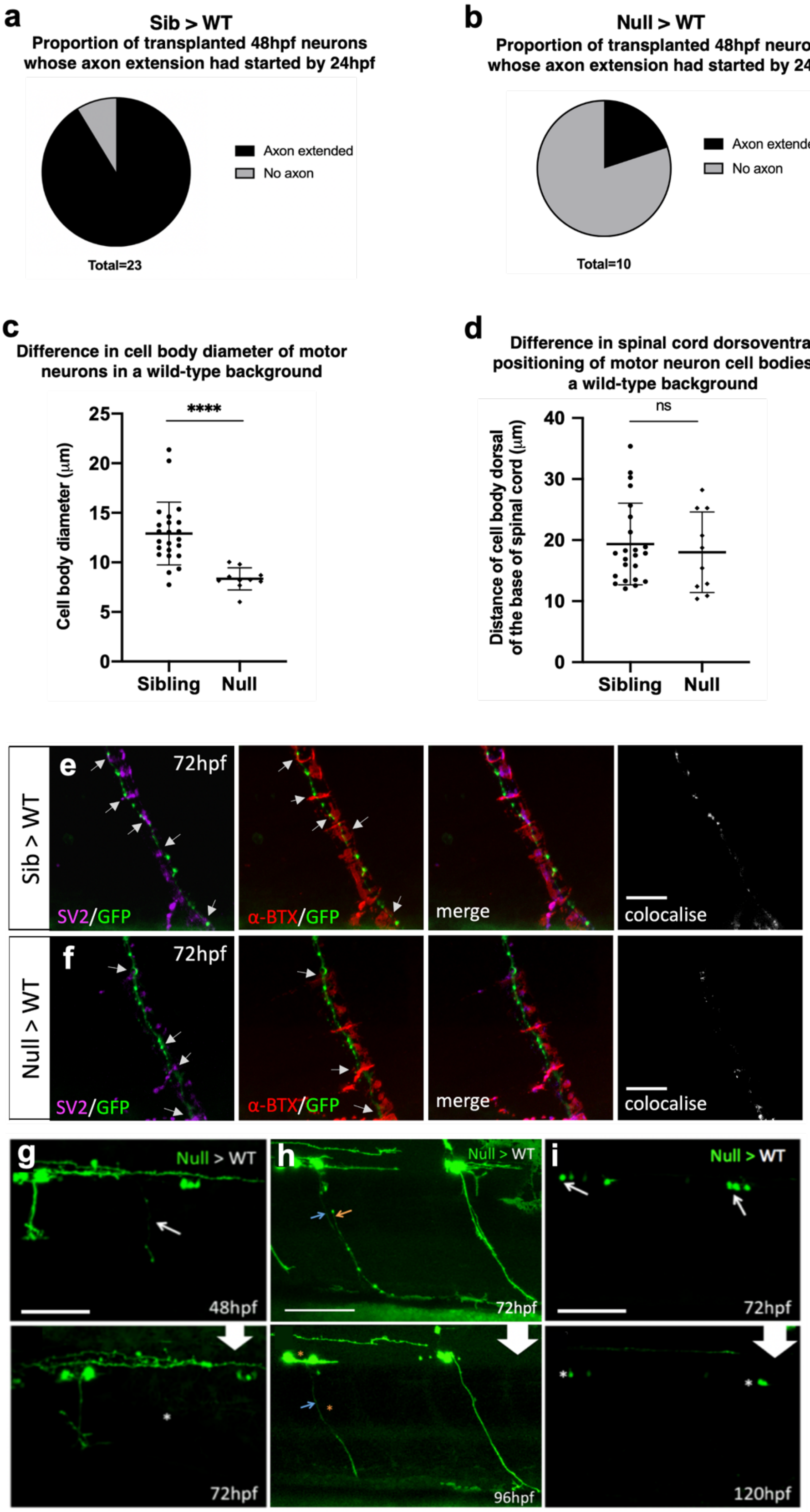

Supplementary Fig. 1: Null motor neurons in a wild-type background show delayed axon extension, branching and synaptogenesis defects, and degenerate at later time points of observation. (**a-b**) Quantification in 48hpf embryos of the proportion of transplanted GFP+ neurons whose axons had extended and exited the spinal cord by 24hpf. (**a**) 87% sibling motor neurons had

initiated axon extension by 24hpf, (Sib>WT: 23cells, 8 hosts (5 donors)). (**b**) Only 20% of null motor neurons had initiated extension by 24hpf and none of these had exited the spinal cord, unlike sibling axons which had all exited (Null>WT: 10 neurons, 3 hosts (3 donors). All sibling and null motor axons at 72hpf were already present at 48hpf. Time-lapse experiments indicated that null axon growth starts at around 40hpf. (**c**) Sibling versus null transplanted motor neuron soma diameter. Sib>WT: 23cells, 8 hosts (5 donors); Null>WT: 10 neurons, 3 hosts, (3 donors). Two-tailed unpaired ttest with Welch's correction: ****=p<0.0001). Means plotted with SD. (**d**) Dorsoventral spinal cord position of sibling and null transplanted motor neuron somas. No difference suggests they do not belong to different motor neuron classes with different axon extension times. Measurements made from the notochord/spinal cord boundary to soma centre (Sib>WT: 23cells, 8 hosts (5 donors); Null>WT: 10 neurons, 3 hosts, (3 donors); Two-tailed unpaired ttest with Welch's correction: (p=0.598). Mean plotted with SD. (**e-f**) Confocal z-projections (10µm), of transplanted *sfpq*; tg(mnx1:GFP) sibling (**e**) & null (**f**) motor neuron distal axons in wild-type hosts. Scale bars 10µm. Arrows show co-localising pre- and post-synaptic markers. Automated detection indicates points of SV2 and α-BTX overlap (mature synapses). (**g-i**) Confocal z-projections (100µm) showing degenerating null motor neurons in a wild-type background - observed each time the experiment was performed but not in siblings. Experiment repeated at least three times. Scale bars 50µm. (**g**) Null axon (arrow) disappears by 72hpf (bottom, asterisk). (**h**) Two null motor neuron axons (blue & orange arrows) with one exhibiting fragmentation (orange arrow). One day later, (**h**, lower), only one motor neuron and axon remain (blue arrow). Asterisk indicates the missing neuron. (**i**) Some null motor neuron somas show no neuronal projections (arrows) at any point during observation and often disappear (lower, asterisks). Source data are provided as a Source Data file.

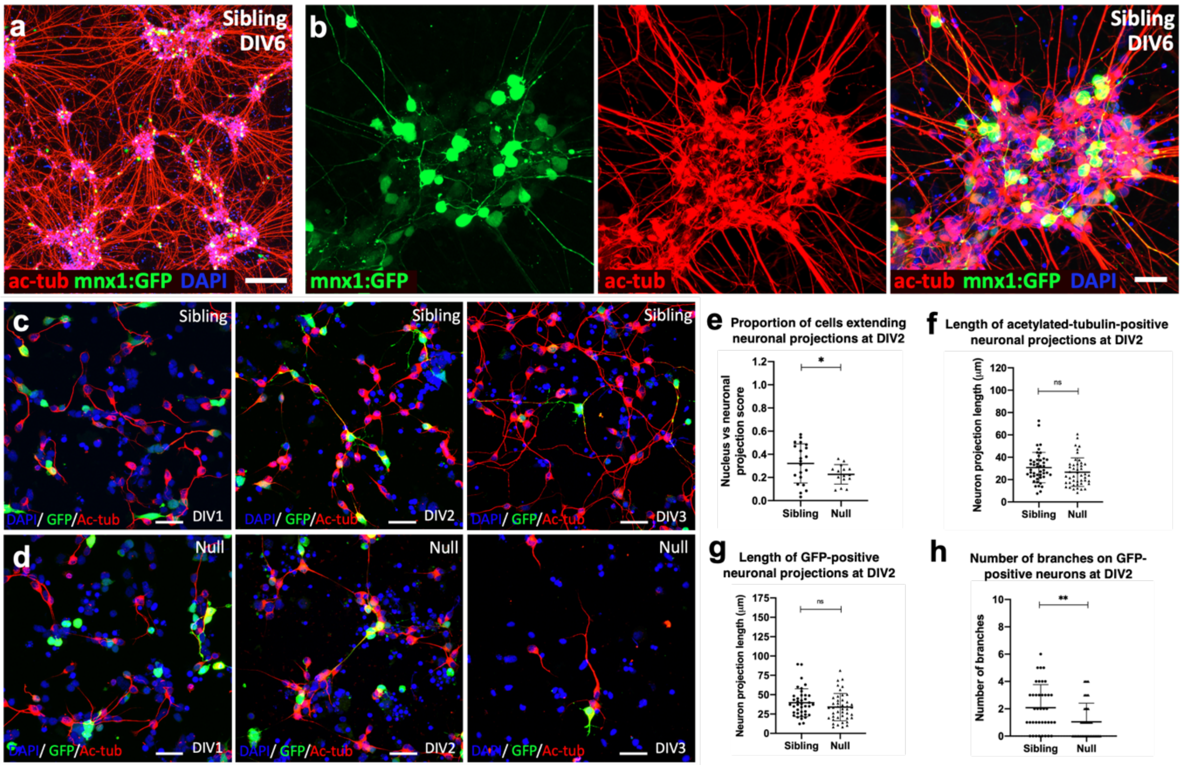

Supplementary Fig. 2: SFPQ-deprived zebrafish primary neurons extend transient neurites *in vitro.* (**a**) Confocal z-projections (20µm) of DIV6 primary cell cultures from dissociated *sfpq,*

tg(mnx1:GFP) embryos. Neurites are immunolabelled using acetylated-tubulin antibody (red). DAPI labels nuclei (blue). Scale bar 100µm. (**b**) High magnification images of cultures described in (**a**). Scale bar: 20µm**.** (**c-d**) Confocal images of sibling (**c**) and null (**d**) primary cell cultures between DIV1-DIV3. Immunolabelling of acetylated-tubulin (red) allowed visualisation of neurites. GFP was targeted to visualise motor neurons. Cell nuclei are labelled by DAPI (blue). Scale bars: 20µm. (**e**) Quantification of the proportion of DAPI stained nuclei associated with acetylated-tubulin-positive neurites in sibling versus null cultures at DIV2, *=p:0.035. n = Sibling: 21 replicates; Null: 15 replicates. N = 3 experiments. Two-tailed unpaired ttest with Welch's correction. Mean plotted with SD. (**f**) Quantification of sibling versus null acetylated-tubulin-positive neurite length at DIV2, p=0.41. n = Sibling: 45 neurites; Null: 45 neurites; 3 independent experiments**.** Two-tailed unpaired ttest with Welch's correction. Mean plotted with SD. (**g**) Quantification of sibling versus null, GFP-positive neurite length in primary cultures at DIV2, p = 0.15. n = Sibling: 39 neurites; Null: 43 neurites; 3 independent experiments. Two-tailed unpaired ttest with Welch's correction. Mean plotted with SD. (**h**) Difference in branch number on sibling and null GFP-positive neurites at DIV2, *=p:0.004. n = Sibling: 39 neurites; Null: 43 neurites; 3 independent experiments. Two-tailed unpaired ttest with Welch's correction. Mean plotted with SD. Source data are provided as a Source Data file.

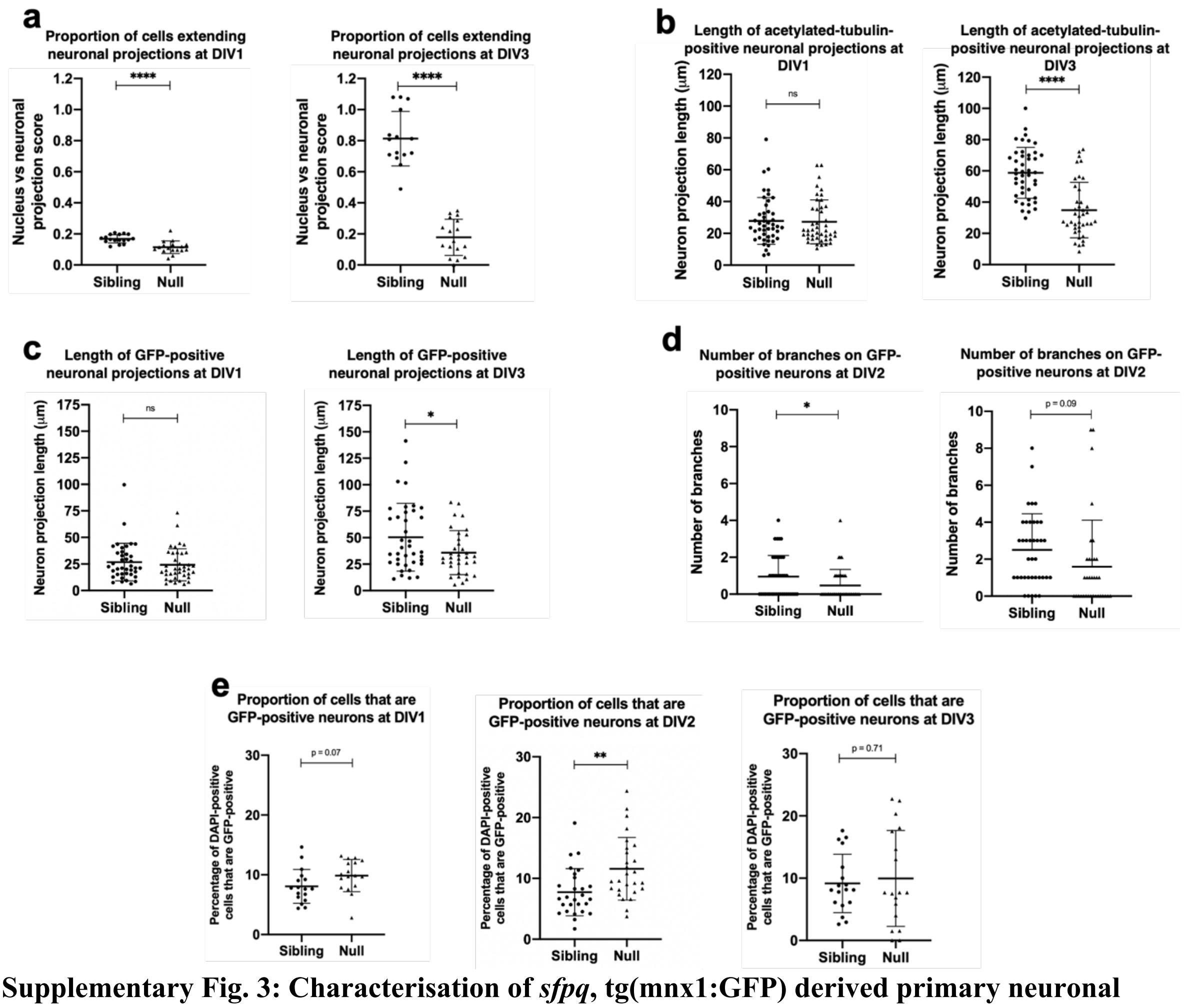

Supplementary Fig. 3: Characterisation of *sfpq*, tg(mnx1:GFP) derived primary neuronal cultures between DIV1-3. (**a**) Quantification of the proportion of DAPI stained nuclei extending acetylated-tubulin-positive axons in sibling versus null cultures at DIV1 (****=p:0.00006. n = Sibling: 17 replicates, Null: 17 replicates. N=3 independent experiments) and DIV3 (****=p:3.7E-13. n = Sibling: 15 replicates, Null: 17 replicates. N= 3 independent experiments). Two-tailed Unpaired

ttest performed with Welch's correction. Mean plotted with SD. (**b**) Quantification of sibling versus null axon lengths by measuring acetylated-tubulin staining at DIV1 (p=0.84. n = Sibling: 45 neurites, Null: 44 neurites. Three independent experiments) & DIV3 (****=p:8.2E-09. n = Sibling: 44 neurites, Null: 40 neurites. Three independent experiments). Two-tailed unpaired ttest with Welch's correction. Mean plotted with SD. (**c**) Quantification of sibling and null GFP-positive neurite lengths at DIV1 (p=0.49. n = Sibling: 39 neurites, Null: 38 neurites. Three independent experiments) & DIV3 (*=p:0.024. n = Sibling: 38 neurites, Null: 34 neurites. Three independent experiments). Two-tailed unpaired ttest with Welch's correction. Mean plotted with SD. (**d**) Quantification of the number of branches exhibited by sibling and null GFP-positive neurites in cultures from *sfpq*, tg(mnx1:GFP) embryos at DIV1 (*=p:0.043. n = Sibling: 39 neurites, Null: 38 neurites. Three independent experiments) & DIV3 (p=0.09. n = Sibling: 38 neurites, Null: 34 neurites. Three independent experiments). Two-tailed unpaired ttest with Welch's correction. Mean plotted with SD. (**e**) Quantification of the percentage of DAPI stained nuclei which were also GFP-positive in sibling and null cultures at DIV1 (p=0.07. n = Sibling: 17 replicates, Null: 17 replicates. Three independent experiments), DIV2 (**=p:0.0031. n = Sibling: 27 replicates, Null: 27 replicates. Three independent experiments), & DIV3 (p=0.71. n = Sibling: 18 replicates, Null: 17 replicates. Three independent experiments) derived from *sfpq*, tg(mnx1:GFP) embryos. GFP-positive cells are mostly motor neurons, and some ventral interneurons. Two-tailed unpaired ttest with Welch's correction. Mean plotted with SD. Source data are provided as a Source Data file.

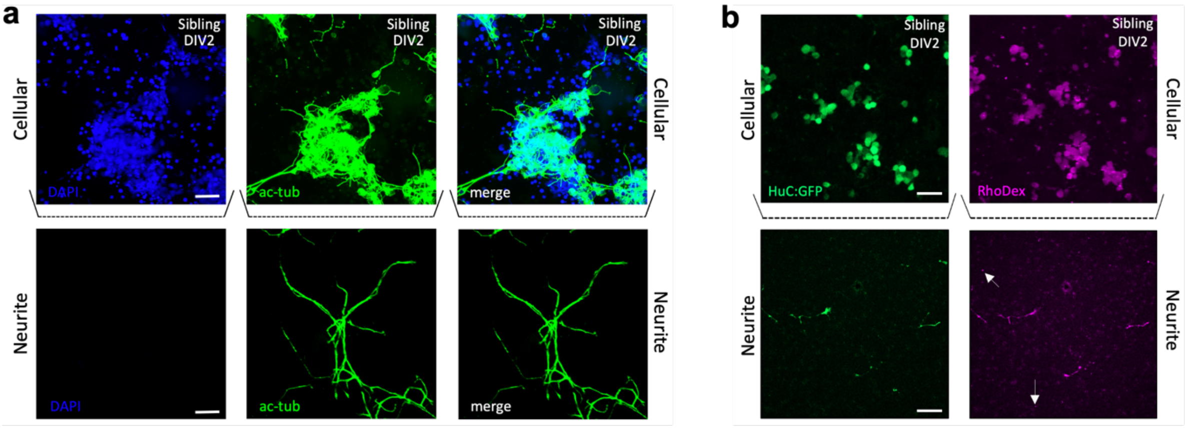

c

GO-term	Description	Count in network	Strength	False discovery rate
GO:0005829	Cytosol	56 of 3167	0.27	0.00078
GO:0030117	Membrane coat	8 of 94	0.95	0.0011
GO:0030424	Axon	19 of 637	0.5	0.0023
GO:0030120	Vesicle coat	6 of 55	1.06	0.0031
GO:0030132	Clathrin coat of coated pit	4 of 17	1.4	0.0045
GO:0030135	Coated vesicle	10 of 215	0.69	0.0059
GO:0043005	Neuron projection	30 of 1458	0.34	0.0066
GO:0030426	Growth cone	9 of 182	0.72	0.0078
GO:0030662	Coated vesicle membrane	7 of 107	0.84	0.0089
GO:0030118	Clathrin coat	5 of 48	1.04	0.0113
GO:0030133	Transport vesicle	12 of 351	0.56	0.0125
GO:0098805	Whole membrane	27 of 1331	0.33	0.0133
GO:0030666	Endocytic vesicle membrane	5 of 61	0.94	0.025
GO:0012507	ER to Golgi transport vesicle membrane	4 of 35	1.08	0.0294
GO:0030127	COPII vesicle coat	3 of 16	1.3	0.0412
GO:0014069	Postsynaptic density	12 of 411	0.49	0.0412
GO:0036464	Cytoplasmic ribonucleoprotein granule	8 of 205	62	0.0479
GO:0030659	Cytoplasmic vesicle membrane	13 of 493	0.45	0.0485

d

Cellular		Neurite	
RNase_MRP	ppib	si:ch211-195b11.4	**gnao1a**
RNaseP_nuc	mdka	elavl4	ddx3b
hbbe1.3	**snoU85**	h3f3b.1	arl8a
**SNORA73**	scarb2a	klhl3	rab14
**SNORA73**	ctsla	hnrnpaba	**mapta**
hbae3	sdc4	**stmn2b**	gnb1a
HBZ	atp2a1	gnb1b	dnaja2l
hbbe3	scg2b	nova2	G3BP2 (1 of many)
**SNORA73**	si:dkey-102m7.3	**mri1**	si:ch73-46j18.5
CR385078.3	cd81a	pbx4	rnf38
RNaseP_nuc	**U4**	**mast2**	**camk2g1**

e

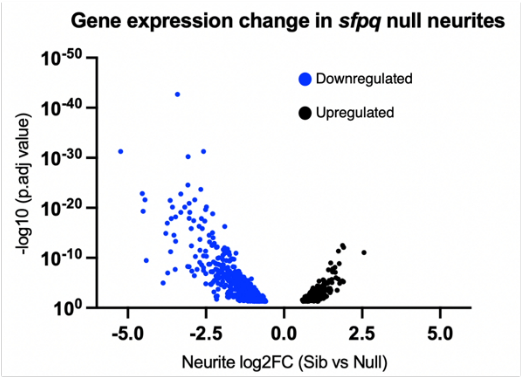

f

GO term	Cellular compartment (GO)	Count in network	Strength	False discovery rate
GO:0030424	Axon	28 of 637	0.59	6.13E-07
GO:0120025	Plasma membrane bounded cell projection	56 of 2292	0.33	8.79E-06
GO:0042995	Cell projection	57 of 2421	0.32	2.00E-05
GO:0043005	Neuron projection	39 of 1458	0.37	0.00011
GO:0005856	Cytoskeleton	54 of 2385	0.3	0.00011
GO:0005829	Cytosol	65 of 3167	0.26	0.00019
GO:0030117	Membrane coat	9 of 94	0.93	0.00023
GO:0030120	Vesicle coat	7 of 55	1.05	0.00047
GO:0045202	Synapse	37 of 1493	0.34	0.00071
GO:0150034	Distal axon	14 of 293	0.62	0.00078
GO:0030426	Growth cone	11 of 182	0.73	0.00085
GO:0030118	Clathrin coat	6 of 48	1.04	0.0018
GO:0030132	Clathrin coat of coated pit	4 of 17	1.32	0.0045
GO:001 4069	Postsynaptic density	15 of 411	0.51	0.0058
GO:0012506	Vesicle membrane	17 of 515	0.46	0.0062
GO:0036477	Somatodendritic compartment	22 of 803	0.38	0.0082
GO:0120111	Neuron projection cytoplasm	6 of 68	0.89	0.0083
GO:0043194	Axon initial segment	4 of 24	1.17	0.009
GO:0030175	Filopodium	7 of 104	0.77	0.009
GO:0030135	Coated vesicle	10 of 215	0.61	0.009
GO:0030133	Transport vesicle	13 of 351	0.51	0.009
GO:0030659	Cytoplasmic vesicle membrane	16 of 493	0.46	0.009
GO:0030425	Dendrite	18 of 593	0.43	0.009
GO:1904115	Axon cytoplasm	5 of 51	0.94	0.0129
GO:0030125	Clathrin vesicle coat	4 of 28	1.1	0.0134
GO:0030027	Lamellipodium	10 of 232	0.58	0.0134
GO:0005905	Clathrin-coated pit	6 of 93	0.75	0.0247
GO:0098858	Actin-based cell projection	9 of 213	0.57	0.0262
GO:0000932	P-body	6 of 95	0.75	0.0265
GO:0031252	Cell leading edge	14 of 460	0.43	0.0269
GO:0098794	Postsynapse	19 of 749	0.35	0.0306
GO:0031209	SCAR complex	3 of 17	1.19	0.0352
GO:0043025	Neuronal cell body	13 of 423	0.43	0.0352
GO:0098805	Whole membrane	28 of 1331	0.27	0.0375
GO:0044304	Main axon	5 of 70	0.8	0.0378
GO:0032433	Filopodium tip	3 of 18	1.17	0.0381
GO:0070382	Exocytic vesicle	9 of 231	0.54	0.0385
G0:0016513	Core-binding factor complex	2 of 4	1.64	0.0421
GO:0030131	Clathrin adaptor complex	3 of 19	1.14	0.0421

Supplementary Fig. 4: Primary cells cultured in transwell inserts for separation of cellular and neurite tissues. (a) Cells from dissociated zebrafish embryos following 2-days of culture in a transwell insert. Scale bar 20µm. Cellular/upper surface: Nuclei labelled by DAPI (blue) indicate cell somas are restricted to this side of the membrane. Many axons labelled by acetylated-tubulin (green) are also observed here. Neurite/lower surface: No nuclei here indicate that no somas pass through 1µm pores. Neurites emerge on the underside and continue their extension after passing through 1µm pores. Experiment repeated three times. (**b**) Transwell membrane after primary cells labelled with rhodamine dextran from tg(HuC:GFP) embryos (in which all neurons are GFP+) have been cultured for 2-days. The vast majority of tissue in the neurite compartment is rhodamine-positive and GFP-positive, with minimal rhodamine-positive and GFP-negative tissue (arrows) observed, indicating a

high degree of purity. Experiment repeated three times. Scale bar 20µm. (**c**) Table showing the cellular compartment gene ontology terms for transcripts enriched in the null neurite compartment, indicating the tissue occupying this compartment is predominantly neurites. (**d**) Most highly expressed transcripts in sibling cellular (left) and neurite (right) compartments. Cellular transcripts include those coding for nuclear factors such as small nucleolar and small nuclear RNAs. Neurite transcripts coding for neuron projection related proteins and include microtubule constituents and microtubule associated proteins. (**e**) Comparing gene expression in sibling and null neurite transcriptomes (Two-sided Fisher's test, p <0.00001) revealed predominantly downregulation (n=597 downregulated genes, Supplementary data 2 (GO term information in main text); n=221 upregulated genes, Supplementary data 3 (GO term analysis of these genes was uninformative, Supplementary data 9). 62/221 genes (28%; Supplementary data 3) showed more upregulation in neurites than in the cellular compartment (neurite-specific upregulation). (**f**) Table showing cell compartment gene ontology terms for transcripts enriched in the null neurite compartment indicating the tissue occupying it is predominantly neurites.

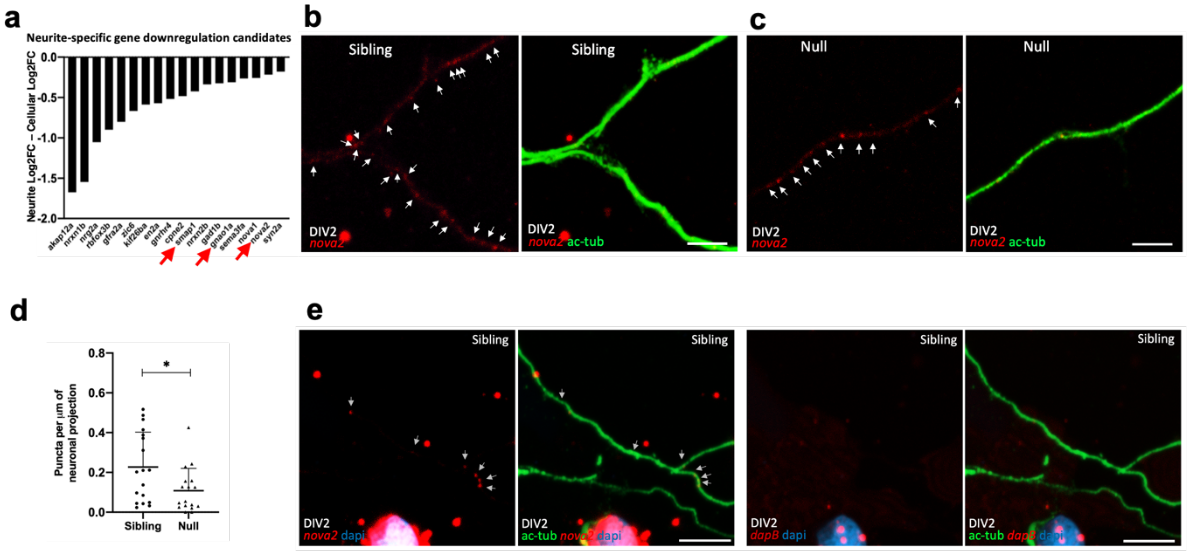

Supplementary Fig. 5: Neurite-specific downregulation of transcripts in absence of SFPQ. (**a**) Extent of neurite-specific gene downregulation in null samples from RNAseq data for 18 genes involved in axonogenesis, taken from the 185 genes in total showing neurite-specific downregulation. Neurite-specific downregulation was identified by calculating the difference in Log2FC values for changes occurring in cellular and neurite compartment comparisons. N = 3 biological replicates for each genotype and cell compartment. (**b-c**) Images of RNAscope labelling of *nova2* puncta (arrows) in sibling (**b**) and null (**c**) cells in culture at DIV2. Scale bar 5µm. (**d**) Quantification of *nova2*-positive puncta in sibling versus null neurites. Each datapoint represents a single neurite (sibling: n=18; null:

n=17). Three independent experiments. Two-tailed Unpaired ttest with Welch's correction, *=p:0.02. Mean plotted with SD. Source data are provided as a Source Data file. (**e**) Confocal images of DIV2 cells plated following dissociation of 24hpf embryos. Arrows indicate *nova2* RNAscope expression puncta in neurites. A probe targeting the bacterial gene *dapb* was used as a negative control to reveal tissue prone to non-specific labelling. This probe was incubated with two biologically independent samples, and no *dapb* puncta were observed in neurites on either occasion. Scale bar 5µm.

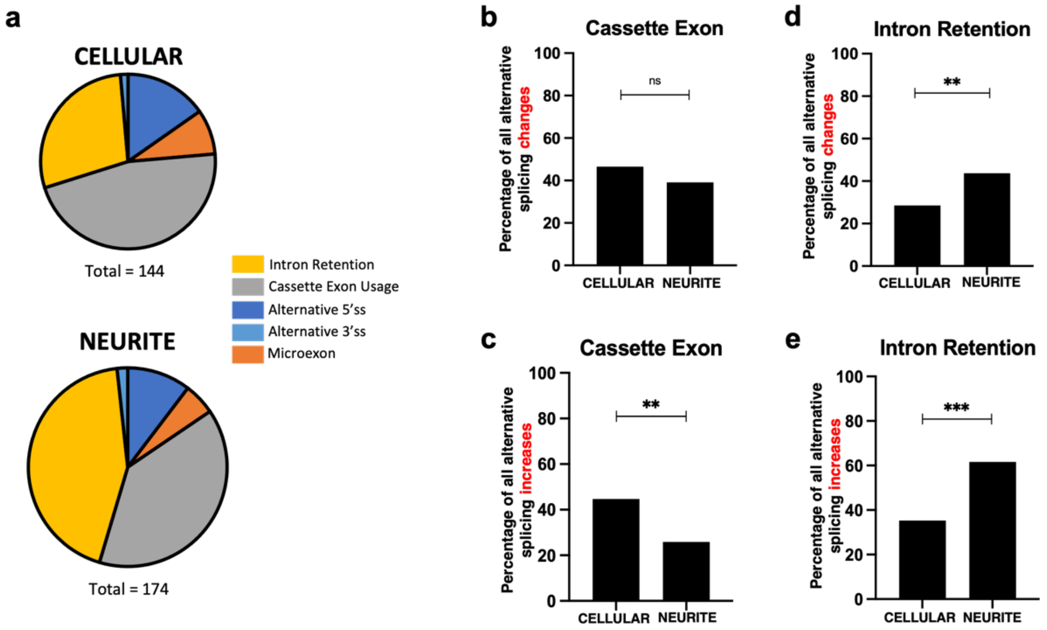

Supplementary Fig. 6: Absence of SFPQ results in neurite-specific increases in intron retention. (**a**) Numbers of events of different splicing types showing statistically significant changes between sibling & null cellular (top) and neurite (bottom) samples. The largest number of changes between cellular compartments found, was in cassette exon usage (n=67; two-tailed Chi-Square = 4.5E-19), whilst between neurite compartments, was in intron retention (n=76; two-tailed Chi-Square = 1.4E-26). Changes between sibling and null samples in either direction had a vast-tools MV.[dPsi]_at_0.95 value ≥0.03 in the respective compartment comparison, and <0.03 for the alterative compartment comparison. No threshold for change (percentage of transcripts) or gene expression level were applied. The profile of splicing changes in null cellular is significantly different from neurite samples (two-tailed Chi-Square = 0.07). (**b-e**) Two-tailed Fisher's test analyses of intron retention (**b-c**) and cassette exon (**d-e**) changes shown in (**a**). (**b & d**) Show the proportion of all alternative splicing changes in null samples that are changes in cassette exon usage (exclusion or inclusion, **b**) and intron retention (**d**), and whether the effect is compartment specific (ns = p:0.21 & **=p:0053, respectively). (**c & e**) Show the extent to which increases in cassette exon usage/inclusion (**c**) and intron retention (**e**) contribute to all alternative splicing increases in null samples and show the compartment specificity of the phenotype (**=p:0.007 and ***=p:0.0003, respectively).

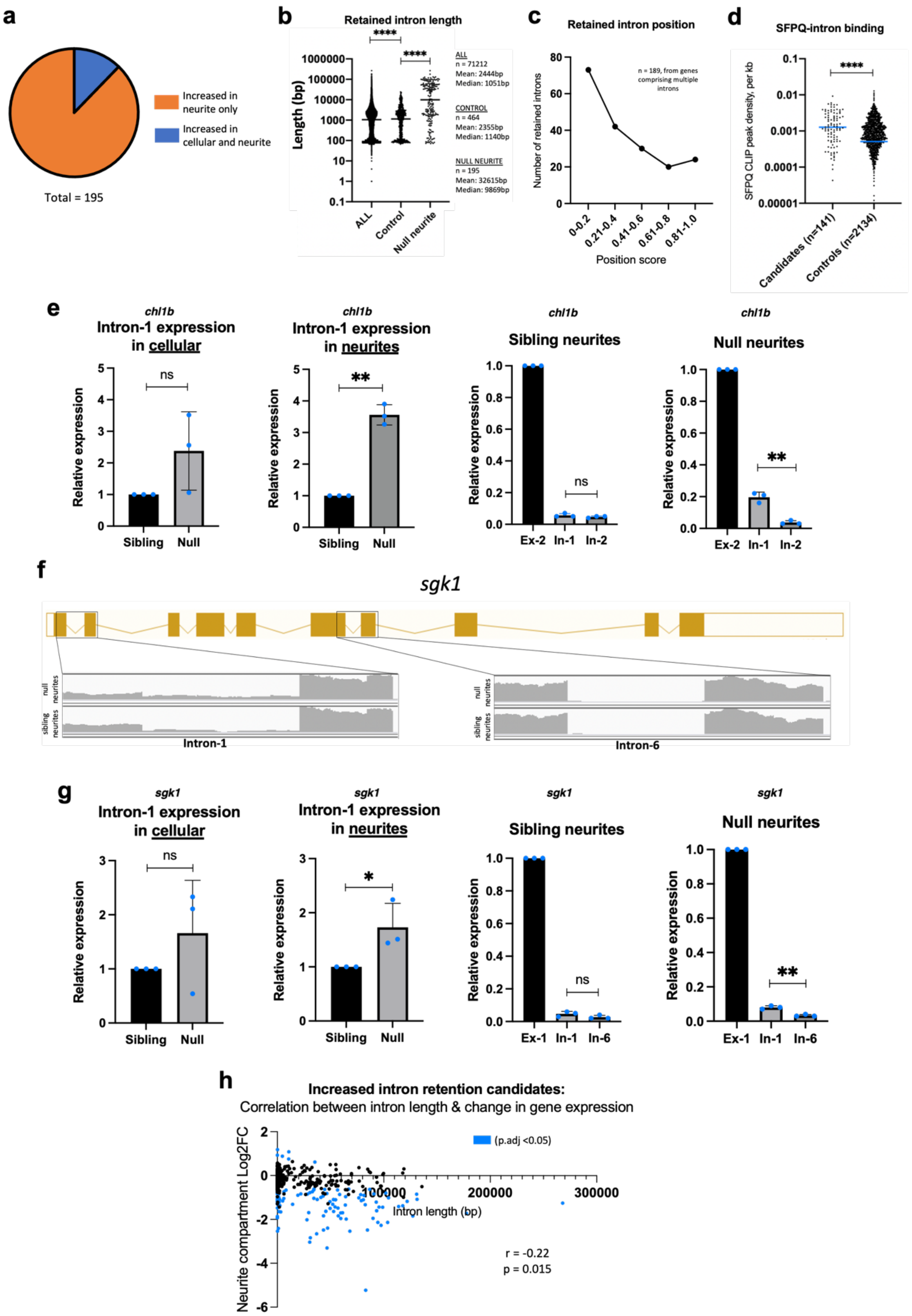

Supplementary Fig. 7: Validation of increased intron retention in neurites of SFPQ-depleted neurons. (**a**) Chart showing neurite-specificity of 195 increased intron retention events revealed by vast-tools. Candidates needed to be retained in at least 10% more of total gene transcripts in null neurites. Statistically significant changes (cellular and neurite comparisons) required an MV.[dPsi]_at_0.95 value ≥ 0.01. 195 introns were significantly changed: 24 increasingly retained in the cellular comparison, 171 (88%) were neurite-specific. (**b**) Lengths of introns showing increased

retention compared to all introns in the analysis, and controls (non-retained introns from the same genes as affected introns). Two-tailed Wilcoxon-signed rank tests, ****=p<0.0001. (**c**) Positional information for the 195 retained introns that come from multiple intron genes. (**d**) CLIP peak density scores for mouse homologues of the 195 introns in (**a**; n=141) and control non-retained introns (n = 2134) from the same genes. Two-sided Wilcoxon signed-rank test, ****=p<0.0001**.** (**e**) RT-qPCR validation of *chl1b* intron-1 (102kbp long) increased retention in null neurites, and no retention of intron-2 (used as control) in either sibling or null neurites. Three biological replicates indicated by blue dots. Means plotted with SD; two-tailed unpaired ttests with Welch's correction, (left two panels) Intron-1 expression in null, relative to sibling levels, p:0.1937 & **=p:0.0052, respectively; (right two panels) Expression of intron-1 and -2 relative to exon-2 expression, which is set to 1. p:0.2739 & **=p:0.0073, respectively. (**f**) Serum/glucocorticoid regulated kinase 1 (*sgk1*) RNAseq BAM files showing increased intron-1 retention in null neurites, and no retention of control intron-6. (**g**) RT-qPCR validation of *sgk1* increased intron-1 (127bp) retention at DIV2. Three biological replicates indicated by blue dots. Means plotted with SD, two-tailed unpaired ttests; (left two panels) Intron-1 expression relative to exon-1 expression, which is set to 1. p:0.3066 & *= p:0.0462, respectively; (right two panels) Expression of introns are relative to Exon-1 expression, which is set to 1. p:0.1447 & **=p:0.0022, respectively. (**h**) Correlation between retained intron length and change in gene expression in null neurites. Blue datapoints:124 intron-retaining genes exhibiting significant expression changes between sibling and null cellular samples (FDR<0.05). Two-sided Pearson correlation coefficient, r (-0.22) and p (0.015). Source data are provided as a Source Data file.

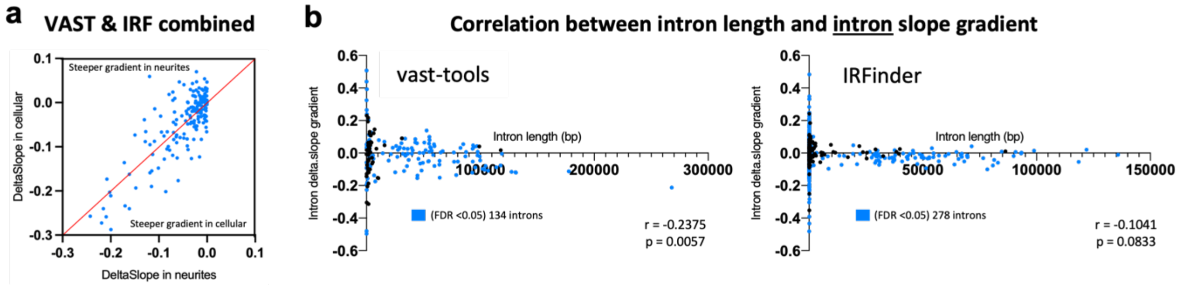

Supplementary Fig. 8: Sloping IR genes in cellular and neurite compartments of SFPQ-depleted neurons. (**a**) Four-way plot showing null cellular and neurite sample gene slope gradients for candidates that have negative slopes in neurites. Negative slope values are generally similar in neurite and cellular samples. Candidates were required to have null cellular and neurite slope gradients with FDR <0.05 in both (n=184). (**b**) Intron length correlates with negative sloping of reads mapped to retained introns in their 5'→3' direction**.**

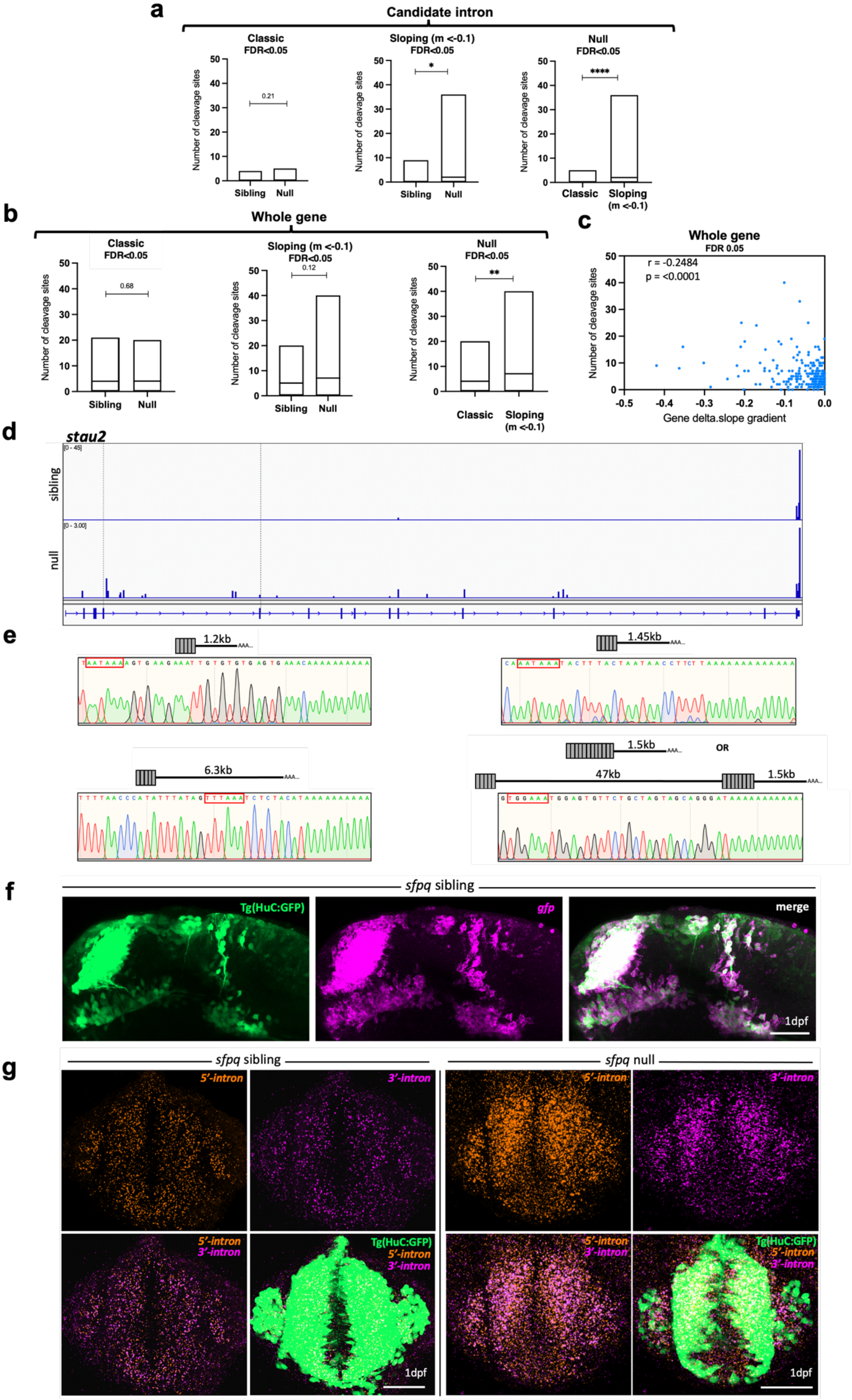
a
Candidate intron
Classic FDR<0.05
Sloping (m <-0.1) FDR<0.05
Null FDR<0.05
Number of cleavage sites
Sibling
Null
Classic
Sloping (m <-0.1)
b
Whole gene
c
Whole gene
FDR 0.05
r = -0.2484
p = <0.0001
Gene delta.slope gradient
d
stau2
sibling
null
e
1.2kb
1.45kb
6.3kb
1.5kb
OR
47kb
f
sfpq sibling
Tg(HuC:GFP)
gfp
merge
1dpf
g
sfpq null
5'-intron
3'-intron

Supplementary Fig. 9: Prematurely terminated, cleaved and polyadenylated IR transcripts localise to neurites in SFPQ-depleted neurons. (**a**) Number of cleavage sites in introns of classic (Left) and sloping (Middle) IR-transcripts in sibling and null (FDR<0.05. Classic, n=107, Sloping, m,<-0.1, n=37). (Right): comparison of the number of cleavage sites in introns of classic and sloping IR-transcripts in null samples. Floating bar plots where the middle represents the median and the upper and lower limits represent the maximum and minimum values. Two-sided Mann-Whitney tests. (Left) p=0.21, (Middle) *=p:0.01 (Right) ****=p:<0.0001. (**b**) Number of cleavage sites along the entire length of classic and sloping genes and how these differ in sibling and null (FDR<0.05. Classic, n=107, Sloping, m,<-0.1, n=37). (Right) Comparison of cleavage site number in classic and sloping genes in null samples. Floating bar plots where the middle represents the median and the upper and lower limits represent the maximum and minimum values. Two-sided Mann-Whitney tests. (Left) p=0.68, (Middle) p=0.12 (Right) **=p:0.0015. (**c**) Correlation between gene slope gradient and the number of cleavage sites across the whole length of the associated genes in null. Two-tailed Pearson correlation coefficient, r: -0.2484, p<0.0001 (n=247)**.** (**d**) 3'mRNAseq read clusters along *stau2* in sibling (top) and null (bottom) 24hpf whole embryo samples, indicating sites of transcript cleavage and polyadenylation. Peak height indicates relative proportion of *stau2* transcripts using that cleavage site. Dashed lines demarcate retained intron-5. Black arrows indicate points of transcript termination targeted for 3'RACE in (e). (**e**) 3'RACE validation of *stau2* 3'mRNAseq results in (**d**), verifying the localisation of PreT-IR transcript isoforms to null neurites. Sequencing traces from 3'RACE products show terminal intronic sequences containing polyadenylation site consensus sequences (red boxes), and the start of the PolyA tail. (**f-g**) Confocal z-projections of 1dpf *sfpq;* tg(HuC:GFP) sibling & null embryos. Experiment repeated three times showing the same results. (**f**) Lateral view, anterior to the left, z-projections (50µm) of embryos after targeting *gfp* mRNA, indicating the protocol achieves high target specificity with minimal background signal. (**g**) Frontal z-projections (20µm) of embryos after targeting *igdcc3* intron-2 IR and PreT-IR transcripts with probe sets targeting the 5'-most 10kb (red) and 3'-most 10kb (magenta) of intron-2. Scale bars 50µm.

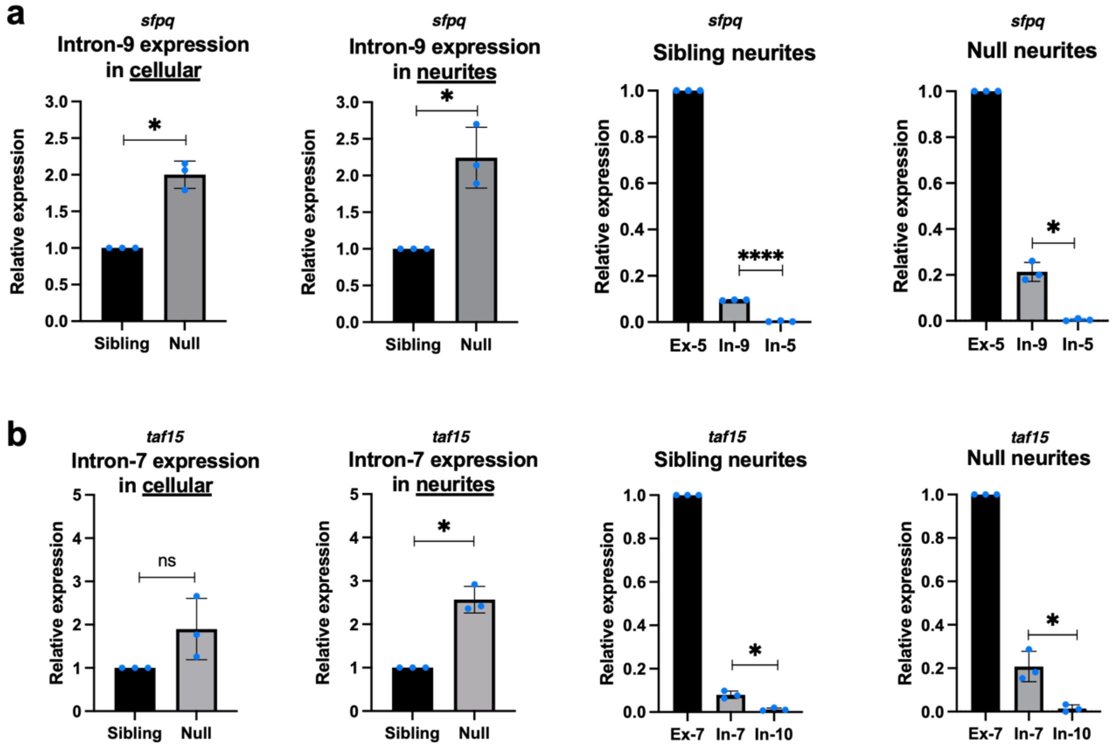

Supplementary Fig. 10: RT-qPCR validation of increased retention of ALS-associated introns in neurites of SFPQ-depleted neurons. (**a**) RT-qPCR validation of *sfpq* intron-9 and intron-5 expression levels. Intron expression normalised to exon-5 expression, which is set to 1. Three biological replicates. Results for individual replicates are indicated by blue dots. Means plotted with SD. Two-tailed unpaired ttests with Welch's correction, (left two panels) Intron-9 expression in null, relative to sibling levels, *= p: 0.0115 & * = p: 0.0352, respectively; (right two panels) Expression of intron-9 and intron-5 relative to exon-5 expression for each sample. ****= p<0.0001 & *= p: 0.0122, respectively. (**b**) RT-qPCR validation of *taf15* intron-7 and control intron-10 levels. Intron expression is normalised to exon-7 expression, which is set to 1. Three biological replicates. Results for individual replicates are indicated by blue dots. Means plotted with SD. Two-tailed unpaired ttests with Welch's correction, (left two panels) Intron-7 expression in null, relative to sibling levels, p: 0.1597 & * = p: 0.0126, respectively; (right two panels) Expression of intron-7 and intron-10 relative to exon-7 expression for each sample. *= p: 0.0119 & *= p: 0.0354, respectively. Source data are provided as a Source Data file.

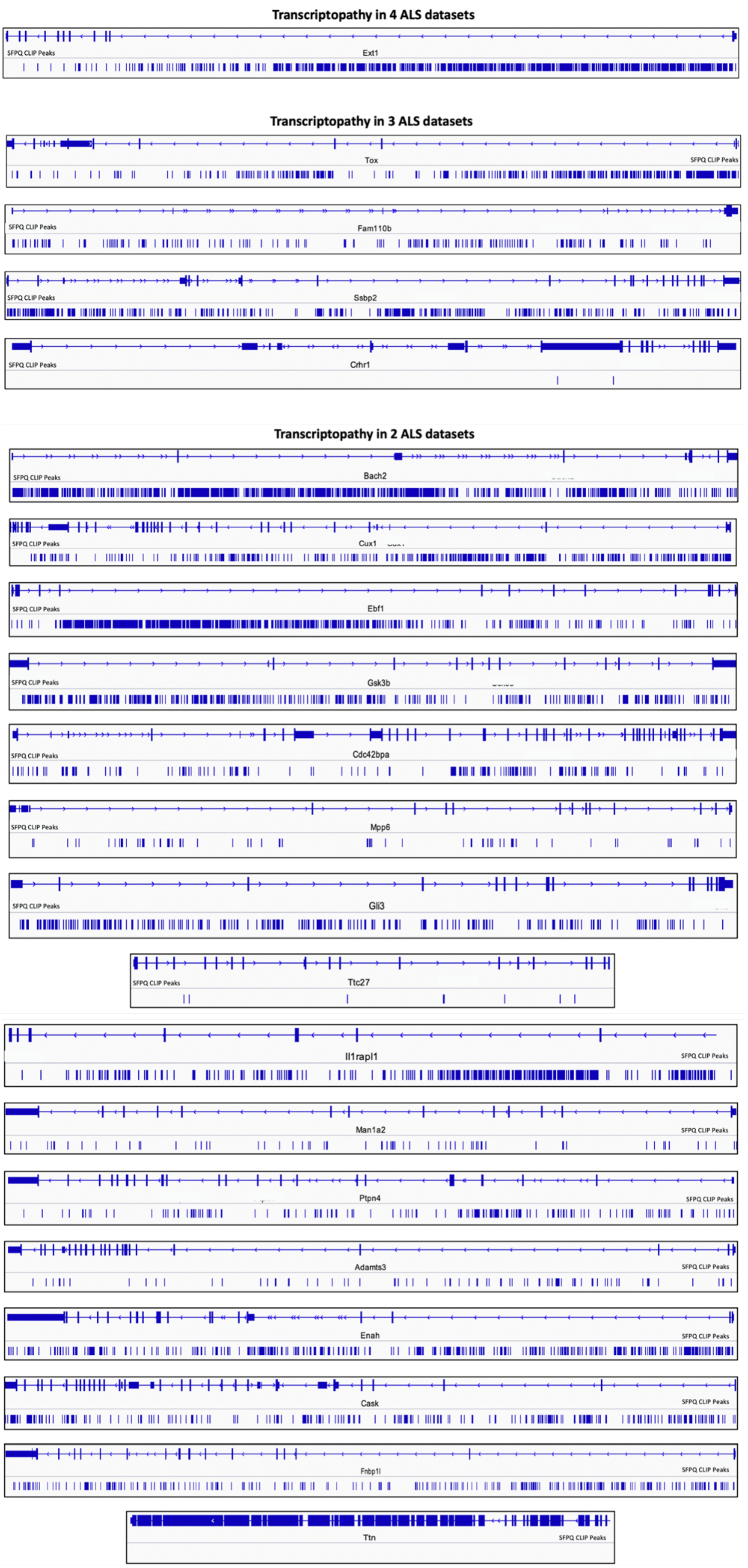
Transcriptopathy in 4 ALS datasets
SFPQ CLIP Peaks
Ext1
Transcriptopathy in 3 ALS datasets
Tox
SFPQ CLIP Peaks
SFPQ CLIP Peaks
Fam110b
SFPQ CLIP Peaks
Ssbp2
SFPQ CLIP Peaks
Crhr1
Transcriptopathy in 2 ALS datasets
SFPQ CLIP Peaks
Bach2
SFPQ CLIP Peaks
Cux1
SFPQ CLIP Peaks
Ebf1
SFPQ CLIP Peaks
Gsk3b
SFPQ CLIP Peaks
Cdc42bpa
SFPQ CLIP Peaks
Mpp6
SFPQ CLIP Peaks
Gli3
SFPQ CLIP Peaks
Ttc27
Il1rapl1
SFPQ CLIP Peaks
Man1a2
SFPQ CLIP Peaks
Ptpn4
SFPQ CLIP Peaks
Adamts3
SFPQ CLIP Peaks
Enah
SFPQ CLIP Peaks
Cask
SFPQ CLIP Peaks
Fnbp1l
SFPQ CLIP Peaks
Ttn
SFPQ CLIP Peaks

Supplementary Fig. 11: Mouse homologues of genes suffering from transcriptopathy in ALS are SFPQ-binding targets. Plots showing SFPQ CLIP binding peaks for mouse homologues of the 21 human genes showing transcriptopathy in ALS. Genes are listed in order of how many of the ALS datasets negative gene sloping was observed.

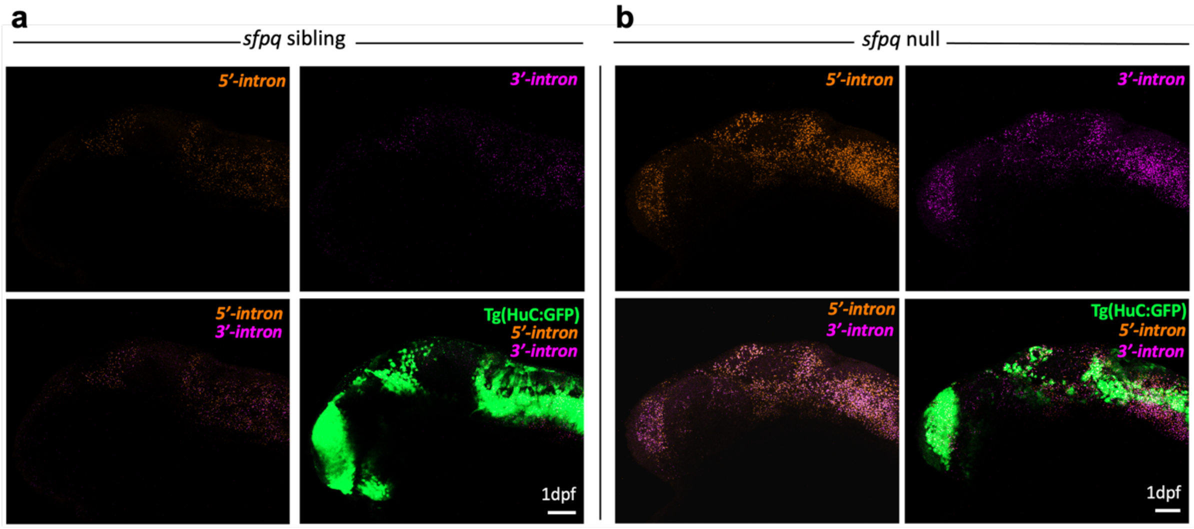

Supplementary Fig. 12: HCR validation of increased *ebf1a* IR and PreT-IR mRNAs in absence of SFPQ. (**a-b**) Confocal z-projections (50µm), lateral view, anterior to the left, of 1dpf *sfpq;* tg(HuC:GFP) sibling (**a**) & null (**b**) embryos following HCR *in situ* hybridisation targeting *ebf1a* intron-6 IR and PreT-IR transcripts with probe sets targeting the 5'-most 10kb of intron-6 (red) and the 3'-most 10kb of intron-6 (magenta). Experiment repeated three times showing the same results. Scale bars 50µm.

Supplementary Tables

gene name	Chromosomal coordinates (danRer10)
bicd1	chr18:12148988-12149519
ccnl1a	chr18:41524050-41526200
cdc37	chrUn_KN149781v1:8333-8518
cntn5	chr18:42550108-42551110
cpsf6	chr4:2201209-2202571
ddx5	chr3:25140624-25141471
foxk1	chr3:40784354-40784620
hnrpdl	chr10:5017211-5018391
insra	chr2:37633836-37635424
ip6k2	chr6:42418044-42418176
katnb1	chr7:51890393-51890479
mgat4a	chr9:7205400-7206073
mgat4b	chr14:16386154-16387273
mgea5	chr13:11864263-11867558
pias2	chr5:40883980-40884051
plcb3	chr7:59988747-59993662
ptbp2a	chr24:30906040-30908582
ptprga	chr11:5088146-5091460
sfpq	chr19:40446206-40452280
srsf1b	chr21:39525930-39526254
srsf5b	chr20:28972734-28975395
taf15	chr15:1874021-1874175
taf15	chr15:1870698-1871229
tars	chr5:43759825-43760707
tnrc6a	chr3:35390725-35391907
u2surp	chr2:26981096-26981618

Supplementary Table 1: ALS-associated intron zebrafish homologues changing in null neurites. Chromosomal coordinates of zebrafish homologous sequences to human (hg19) and mouse (mm10) ALS-associated introns in supplementary data 8. Most homologous sequences were identified using LiftOver. Others were identified by manually identifying conserved exons flanking introns.

gene_name	gene_length	source	dataset	ALS.slope	ALS.FDR	Ctrl.slope	Ctrl.FDR	deltaSlope
ADAMTS3	287831	hg19	Kapeli	-0.1430921	0.04357726	-0.0780222	0.07795231	-0.0650699
ADAMTS3	287831	hg19	Tam	-0.3665839	0.02645913	-0.269496	0.02810459	-0.0970879
Bach2	347305	mm10	Fratta	0.10422769	0.01367517	0.16561113	0.00239279	-0.0613834
BACH2	370380	hg19	Tam	-0.4147708	0.02368988	-0.2820375	0.03373779	-0.1327333
CASK	408530	hg19	dbGAP	-0.3197458	0.0004129	-0.2452457	0.0006924	-0.0745001
CASK	408530	hg19	Tam	-0.5063408	0.0064886	-0.3894378	0.00269847	-0.116903
CDC42BPA	328610	hg19	dbGAP	-0.2097594	0.01058325	-0.1572581	0.01898697	-0.0525013
CDC42BPA	328610	hg19	Tam	-0.3660673	0.04287814	-0.3087493	0.01776459	-0.057318
CRHR1	213928	hg19	dbGAP	-0.6224442	0.00091456	-0.5144334	0.00045762	-0.1080108
CRHR1	213928	hg19	Kapeli	-0.6574627	0.00064554	-0.3065992	0.00033717	-0.3508635
CRHR1	213928	hg19	Tam	-0.6741939	0.00011094	-0.4854412	0.00014787	-0.1887528
CUX1	468291	hg19	dbGAP	-0.3539721	0.04513972	-0.2770637	0.04262705	-0.0769084
CUX1	468020	hg19	dbGAP	-0.3539721	0.04513972	-0.2770637	0.04262705	-0.0769084
CUX1	468291	hg19	Tam	-0.7480618	0.00524746	-0.5454478	0.00543187	-0.202614
CUX1	468020	hg19	Tam	-0.7480618	0.00524746	-0.5454478	0.00543187	-0.202614
EBF1	403842	hg19	Kapeli	-0.2048966	0.0293606	-0.1016818	0.16044431	-0.1032147
EBF1	403842	hg19	Tam	-0.4259566	0.01827206	-0.2961014	0.01637938	-0.1298552
ENAH	166308	hg19	dbGAP	-0.28601	0.00721496	-0.202885	0.01503054	-0.083125
ENAH	166308	hg19	Tam	-0.6260931	0.00080382	-0.4518151	0.00079978	-0.174278
EXT1	317364	hg19	dbGAP	-0.4210512	1.76E-06	-0.3521093	1.21E-06	-0.0689419
EXT1	317364	hg19	Kapeli	-0.4346188	1.27E-06	-0.3615271	9.72E-06	-0.0730917
EXT1	317364	hg19	Prudencio	-0.3350004	0.01278941	-0.0269035	0.61654063	-0.3080969
EXT1	317364	hg19	Tam	-0.8883469	1.61E-06	-0.6337064	1.61E-06	-0.2546405
FAM110B	209771	hg19	dbGAP	-0.2886052	0.00038994	-0.2348322	0.00049553	-0.053773
FAM110B	209771	hg19	Kapeli	-0.3223436	0.0005597	-0.2417645	0.02795265	-0.0805791
FAM110B	209771	hg19	Tam	-0.75965	5.73E-06	-0.5104219	3.13E-06	-0.2492281
FNBP1L	106531	hg19	Kapeli	-0.2668782	0.01227335	-0.2135553	0.01297815	-0.0533229
FNBP1L	106531	hg19	Tam	-0.5362178	7.91E-05	-0.3994781	2.87E-05	-0.1367397
Gli3	266792	mm10	Fratta	0.110264	0.01011365	0.16080444	0.00388117	-0.0505404
GLI3	276922	hg19	Tam	-0.6053877	0.00248824	-0.4508049	0.00215904	-0.1545828
GSK3B	273095	hg19	dbGAP	-0.1736168	0.01853273	-0.1164017	0.0406575	-0.0572151
GSK3B	273095	hg19	Kapeli	-0.2093355	0.03535413	-0.1432123	0.07609995	-0.0661233
IL1RAPL1	1369325	hg19	dbGAP	-0.3980473	5.40E-12	-0.329774	4.69E-12	-0.0682733
IL1RAPL1	1369325	hg19	Tam	-0.8678064	6.71E-13	-0.5651713	4.69E-12	-0.3026352
MAN1A2	161424	hg19	dbGAP	-0.3223128	0.00344607	-0.2720203	0.00367518	-0.0502925
MAN1A2	161424	hg19	Tam	-1.0000002	0.00896601	-0.7724933	0.01053199	-0.2275069
MPP6	120926	hg19	dbGAP	-0.4311177	0.00045512	-0.335422	0.00038	-0.0956956
MPP6	120926	hg19	Tam	-0.7344837	0.00106612	-0.589397	0.00076124	-0.1450867
PTPN4	224188	hg19	dbGAP	-0.2193462	0.00564127	-0.1635961	0.01030999	-0.0557501
PTPN4	224188	hg19	Tam	-0.5501958	0.00781406	-0.4358137	0.00371439	-0.1143821
SSBP2	338777	hg19	dbGAP	-0.3801758	0.00777705	-0.3220598	0.0043679	-0.058116
SSBP2	338777	hg19	Prudencio	-0.330192	0.04517586	-0.0215085	0.66360558	-0.3086835
SSBP2	338777	hg19	Tam	-0.7904475	0.01101675	-0.5787241	0.00959026	-0.2117234
TOX	313791	hg19	Kapeli	-0.3401924	3.64E-05	-0.222814	0.00014787	-0.1173785
TOX	313791	hg19	Prudencio	-0.1688098	0.02493083	0.01585519	0.74122262	-0.184665
TOX	313791	hg19	Tam	-0.7613675	8.86E-08	-0.5455356	9.07E-08	-0.2158319
TTC27	193020	hg19	Kapeli	-0.1891719	0.00228251	-0.0872767	0.11594383	-0.1018952
TTC27	193020	hg19	Tam	-0.5049881	0.00011203	-0.3166787	8.64E-05	-0.1883094
Ttn	278568	mm10	Bozzoni	0.41634782	2.77E-11	0.6429872	6.27E-13	-0.2266394
Ttn	278568	mm10	Fratta	0.08756394	8.42E-05	0.21186637	0.00103232	-0.1243024

Supplementary Table 2: Transcriptopathy in ALS candidate genes. 21 long genes that show statistically significant transcriptopathy in two or more of 6 ALS datasets compared to control (FDR <0.05).
