# Supplementary material for: Prematurely terminated intron-retaining mRNAs invade axons in SFPQ null-driven neurodegeneration and are a hallmark of ALS
Source: Nat Commun. 2022 Nov 22;13:6994. doi: 10.1038/s41467-022-34331-4 (PMC9681851; doi:10.1038/s41467-022-34331-4)
Supplement: Supplementary file 3 — Description of Additional Supplementary Files [file 41467_2022_34331_MOESM3_ESM.pdf]

Description of Additional Supplementary Files

Supplementary Data 1: Genes enriched in sibling cellular and neurite samples. Genes enriched in sibling cellular compartment compared to sibling neurite compartment (fold change > 1.5, p <0.05). Genes enriched in sibling neurite compartments compared to sibling cellular compartment (fold change > 1.25, p<0.05).

Supplementary Data 2: Gene downregulation in neurites (and neurite-specific). 597 annotated genes expressed more in sibling neurites (FC >1.5) than in null neurites. Genes were filtered to remove those expressed <20 TpM in sibling neurites. 185 genes annotated genes downregulated more in null neurites than in null cellular samples (Log2FC Neurites - Log2FC Cellular = <-0.2).

Supplementary Data 3: Gene upregulation in neurites (and neurite-specific). 221 annotated genes expressed more in null neurites (FC >1.5) than in sibling neurites. Genes were filtered to remove those expressed <20 TpM in sibling neurites. 62 genes annotated genes upregulated more in null neurites than in null cellular samples (Log2FC Neurites - Log2FC Cellular = >0.2).

Supplementary Data 4: Initial splicing analysis. All splicing changes (alternative 5'SS (Alt5), alternative 3'SS (Alt3), cassette exon (CE), microexon (MIC), intron retention (IR)) detected using vast-tools. Changes between sibling and null samples required MV.[dPsi]_at_0.95 = ≥0.03 in the respective compartment comparison, and MV.[dPsi]_at_0.95 = <0.03 for the other compartment comparison. No threshold for extent of change (percentage of total transcripts showing the splicing change in question), or gene expression level were applied.

Supplementary Data 5: vast-tools and IRFinder intron retention. 195 introns more retained and 35 introns less retained in null neurites than in sibling neurites (>10% change in either direction of transcripts being intron-retaining and MV.[dPsi]_at_0.95 value ≥0.01). In IRFinder analyses, 446 IR events showed increases (> 0.1 increase in IR Ratio) p<0.05, and 86 events showed decreases (< -0.1 increase in IR Ratio) p<0.05 in retention in null neurites when compared to sibling counterparts.

Supplementary Data 6: Genes whose transcripts are preferentially translated in neurites. Ribosomal footprint (Riboseq) data from rat hippocampus indicates that orthologues of 154 zebrafish genes, whose transcripts showing neurite-specific increases in IR, are preferentially translated in neurites.

Supplementary Data 7: Stable versus negative sloping gene lists. Of the genes identified by vast-tools to exhibit increased intron retention in null neurites, 46 show stable downstream exon expression and 135 intron-retaining genes show negative downstream exon expression. Full candidate lists shown before filtering out those with values whose FDR >0.05. Of the IRFinder identified genes exhibiting increased intron retention in null neurites, 157 show stable downstream exon expression and 252 intron-retaining genes show negative downstream exon expression. Full candidate lists shown before filtering out those with values whose FDR >0.05.

Supplementary Data 8: ALS-associated human and mouse introns. Chromosomal coordinates of human (hg19) and mouse (mm10) introns perturbed in ALS patient samples and models of ALS.

Supplementary Data 9: Enriched Gene Ontology Biological Processes

Supplementary Data 10: Primers
