# Supplementary material for: Prematurely terminated intron-retaining mRNAs invade axons in SFPQ null-driven neurodegeneration and are a hallmark of ALS
Source: Nat Commun. 2022 Nov 22;13:6994. doi: 10.1038/s41467-022-34331-4 (PMC9681851; doi:10.1038/s41467-022-34331-4)
Supplement: Supplementary file 14 — Reporting Summary [file 41467_2022_34331_MOESM14_ESM.pdf]

nature portfolio

Corresponding author(s): Richard Taylor, Corinne Houart

Last updated by author(s): Oct 16, 2022

Reporting Summary

Nature Portfolio wishes to improve the reproducibility of the work that we publish. This form provides structure for consistency and transparency in reporting. For further information on Nature Portfolio policies, see our Editorial Policies and the Editorial Policy Checklist.

Statistics

For all statistical analyses, confirm that the following items are present in the figure legend, table legend, main text, or Methods section.

n/a	Confirmed	
☐	☒	The exact sample size (*n*) for each experimental group/condition, given as a discrete number and unit of measurement
☐	☒	A statement on whether measurements were taken from distinct samples or whether the same sample was measured repeatedly
☐	☒	The statistical test(s) used AND whether they are one- or two-sided<br>*Only common tests should be described solely by name; describe more complex techniques in the Methods section.*
☐	☒	A description of all covariates tested
☐	☒	A description of any assumptions or corrections, such as tests of normality and adjustment for multiple comparisons
☐	☒	A full description of the statistical parameters including central tendency (e.g. means) or other basic estimates (e.g. regression coefficient) AND variation (e.g. standard deviation) or associated estimates of uncertainty (e.g. confidence intervals)
☐	☒	For null hypothesis testing, the test statistic (e.g. F, t, r) with confidence intervals, effect sizes, degrees of freedom and P value noted<br>*Give P values as exact values whenever suitable.*
☒	☐	For Bayesian analysis, information on the choice of priors and Markov chain Monte Carlo settings
☒	☐	For hierarchical and complex designs, identification of the appropriate level for tests and full reporting of outcomes
☐	☒	Estimates of effect sizes (e.g. Cohen's d, Pearson's r), indicating how they were calculated

Our web collection on statistics for biologists contains articles on many of the points above.

Software and code

Policy information about availability of computer code

Data collection	LightCycler 96 software [Version 1.1.0.1320]; Roche

Data analysis

Relevant computer code is described in the Methods.

The main software used were:
- VAST-TOOLS (Version 2.1.3); (Braunschweig et al., 2014); https://github.com/vastgroup/vast-tools
- IRFinder (version 1.3.1); (Middleton et al., 2017); https://github.com/williamritchie/IRFinder/releases
- FastQC (Version 0.11.8); Andrew, 2010); http://www.bioinformatics.babraham.ac.uk/projects/fastqc/
- HISAT2 (Version 2.1.0); (Kim et al,, 2015); http://ccb.jhu.edu/software/hisat2/index.shtml
- Samtools (Version 1.9); (Li et al., 2009); https://sourceforge.net/projects/samtools/
- GenomicRanges (Version 3.12); (Lawrence et al., 2013); https://bioconductor.org/packages/release/bioc/html/GenomicRanges.html
- R (Version 4.0.3); (RCoreTeam, 2020); https://www.r-project.org/
- edgeR (Version 3.12.1); (Robinson et al., 2010); https://www.bioconductor.org/packages/release/bioc/html/edgeR.html
- IGV (Version 2.4); (Robinson et al., 2011); https://software.broadinstitute.org/software/igv/download
- FIJI (Version 2.0.0-rc-69/1.52); (Schindelin et al., 2012); https://imagej.net/software/fiji/
- GraphPad Prism 9 (https://www.graphpad.com/scientific-software/prism/)

For manuscripts utilizing custom algorithms or software that are central to the research but not yet described in published literature, software must be made available to editors and reviewers. We strongly encourage code deposition in a community repository (e.g. GitHub). See the Nature Portfolio guidelines for submitting code & software for further information.

Data

Policy information about availability of data

All manuscripts must include a data availability statement. This statement should provide the following information, where applicable:
- Accession codes, unique identifiers, or web links for publicly available datasets
- A description of any restrictions on data availability
- For clinical datasets or third party data, please ensure that the statement adheres to our policy

All data generated in this study are provided in the Supplementary Information/Source Data file, or at the accession links below.

- Cellular and neurite transcriptome RNA-seq data from sfpq sibling and null zebrafish lines is available at ArrayExpress, at accession number E-MTAB-11431 (https://www.ebi.ac.uk/arrayexpress/experiments/E-MTAB-11431/).

- 3' mRNA-seq data from sfpq sibling and null zebrafish lines was taken from a study by Gordon et al. (2021). Data is available at accession number E-MTAB-9899 (https://www.ebi.ac.uk/arrayexpress/experiments/E-MTAB-9899/).

- Mouse Sfpq CLIP-seq data was taken from a study by Takeuchi et al. (2018). Data is available at accession number GSE60246 (https://www.ncbi.nlm.nih.gov/geo/query/acc.cgi?acc=GSE60246).

- GRCz10 assembly is available for download at link (https://www.ncbi.nlm.nih.gov/assembly/GCF_000002035.5/).

- Transcriptomics data from ALS models were taken from various studies:
  - Humphrey et al. (2020): GSE147288
  - Prudencio et al. (2015): GSE67196
  - Tam et al. (2019): GSE124439.
  - Brohawn et al. (2016): SRP064478
  - D'Erchia et al. (2017): phs000747 (dbGaP)

Human research participants

Policy information about studies involving human research participants and Sex and Gender in Research.

Reporting on sex and gender	The study did not involve human research participants.
Population characteristics	N/A
Recruitment	N/A
Ethics oversight	N/A

Note that full information on the approval of the study protocol must also be provided in the manuscript.

Field-specific reporting

Please select the one below that is the best fit for your research. If you are not sure, read the appropriate sections before making your selection.

☒ Life sciences ☐ Behavioural & social sciences ☐ Ecological, evolutionary & environmental sciences

For a reference copy of the document with all sections, see nature.com/documents/nr-reporting-summary-flat.pdf

Life sciences study design

All studies must disclose on these points even when the disclosure is negative.

Sample size	No sample-size calculations were performed. Experiments were performed with at least n=3 biological replicates allowing for rigorous statistical analysis to be performed in comparisons made.
Data exclusions	No data was excluded.
Replication	Experiments were reproduced at least once. No experiments failed to reproduce.
Randomization	Sample allocation was random
Blinding	No blinding was performed during data collection and analysis. Morphological differences between sfpq sibling and null embryos and neurons make them easily discernible.

Reporting for specific materials, systems and methods

We require information from authors about some types of materials, experimental systems and methods used in many studies. Here, indicate whether each material, system or method listed is relevant to your study. If you are not sure if a list item applies to your research, read the appropriate section before selecting a response.

Materials & experimental systems

n/a	Involved in the study
☐	☒ Antibodies
☒	☐ Eukaryotic cell lines
☒	☐ Palaeontology and archaeology
☐	☒ Animals and other organisms
☒	☐ Clinical data
☒	☐ Dual use research of concern

Methods

n/a	Involved in the study
☒	☐ ChIP-seq
☒	☐ Flow cytometry
☒	☐ MRI-based neuroimaging

Antibodies

Antibodies used	anti-GFP (Torrey Pines Biolabs Inc, TP401) (1:500),<br>anti-SV2A (Developmental Studies Hybridoma Bank, AB2315387)(1:100),<br>anti-acetylated tubulin (Sigma-Aldrich, T6793)(1:1000),<br>Anti-rabbit IgG (H+L) Alexa Fluor 488 (Invitrogen, A11008)(1:1000),<br>Anti-mouse IgG (H+L) Alexa Fluor 488 (Invitrogen, A11001)(1:1000),<br>Anti-mouse IgG (H+L) Alexa Fluor 568 (Invitrogen, A11004)(1:1000),<br>Anti-mouse IgG (H+L) Alexa Fluor 633 (Invitrogen, A21050)(1:1000),
Validation	The anti-GFP, anti-SV2A, and anti-acetylated tubulin antibodies listed above have been used extensively for immunofluorescent labelling in zebrafish research, including in our previous work (Thomas-Jinu et al., 2017, Neuron).

Animals and other research organisms

Policy information about studies involving animals; ARRIVE guidelines recommended for reporting animal research, and Sex and Gender in Research

Laboratory animals	Zebrafish of the AB wildtype and sfpq-kg41 strains were used, including in tg(mnx1:GFP) and tg(HuC:GFP) backgrounds. Adults were only used for breeding (3 months - 2 years of age). Embryos/larvae were only analysed up to 120 hours post fertilisation.
Wild animals	The study did not include wild animals.

Reporting on sex	None of the findings in the study relate to sex.
Field-collected samples	The study did not involve samples collected from the field.
Ethics oversight	All work using zebrafish was approved by the UK Home Office Animals in Science Regulation Unit in accordance with the Animals (Scientific Procedures) Act 1986, under CH Home Office Project license 70/7577.

Note that full information on the approval of the study protocol must also be provided in the manuscript.
